# Severe manifestations of SARS-CoV-2 in children and adolescents: from COVID-19 pneumonia to multisystem inflammatory syndrome: a multicentre study in pediatric intensive care units in Spain

**DOI:** 10.1186/s13054-020-03332-4

**Published:** 2020-11-26

**Authors:** Alberto García-Salido, Juan Carlos de Carlos Vicente, Sylvia Belda Hofheinz, Joan Balcells Ramírez, María Slöcker Barrio, Inés Leóz Gordillo, Alexandra Hernández Yuste, Carmina Guitart Pardellans, Maite Cuervas-Mons Tejedor, Beatriz Huidobro Labarga, José Luís Vázquez Martínez, Míriam Gutiérrez Jimeno, Ignacio Oulego-Erróz, Javier Trastoy Quintela, Carmen Medina Monzón, Laura Medina Ramos, María Soledad Holanda Peña, Javier Gil-Antón, Clara Sorribes Ortí, José Carlos Flores González, Rosa María Hernández Palomo, Inma Sánchez Ganfornina, Emilia Fernández Romero, María García-Besteiro, Jesús López-Herce Cid, Rafael González Cortés, the Spanish Pediatric Intensive Care Society working group on SARS-CoV-2 infection, María Slöcker Barrio, Amaya Bustinza Arriortua, Jesús López-Herce Cid, Rafael González Cortés, Juan Carlos de Carlos Vicente, Maite Cuervas-Mons Tejedor, Pedro Pablo Oyágüez Ugidos, Iolanda Jordan, Carmina Guitart Pardellans, Sonia Sanchíz Cárdenas, Javier Gil-Antón, Belén Joyanes, Ainhoa Jiménez Olmos, Antonio Rodríguez Núñez, Javier Trastoy Quintela, Alexandra Hernández Yuste, Laura Díaz Munilla, Carlos Solís Reyes, Laura Medina Ramos, David Roca Pascual, Joan Ballcels Ramírez, Mario Sánchez Fernández, Alberto García-Salido, Inés Leóz Gordillo, Montserrat Nieto Moro, Amelia Martínez de Azagra Garde, María Ángeles García Teresa, Corsino Rey Galán, Alfredo Molina Cambra, Manuel González-Ripoll Garzón, Pepe Fernández-Cantalejo Padial, Ignacio Oulego-Erroz, Laia Vega Puyal, Daniel Moreno, Emilia Fernández Romero, María García Besteiro, José Carlos Flores González, Carmen Medina Monzón, Beatriz Huidobro Labarga, Rosa María Hernández Palomo, Cristina Calvo Monge, Francisco Fernández, Nieves González, Lorena Bermúdez Barrezueta, Cesar Villa Francisco, Ana Abril Molina, Mónica Valeron, Ramón Hernández Rastrollo, Sylvia Belda Hofheinz, Manuel Gijón Mediavilla, José Luis Vázquez Martínez, Manuel Frias, Raúl Montero Yéboles, Juan Ignacio Muñóz Bonet, María Velázquez, Inma Sánchez Ganfornina, Antonio Pérez Iranzo, David Lozano, Clara Sorribes Ortí, María Soledad Holanda Peña, Miriam Gutiérrez Jimeno

**Affiliations:** 1 Hospital Infantil Universitario Niño Jesús, Madrid, Spain; 2 Hospital Universitario Son Espases, Palma, Spain; 3 Hospital Universitario 12 de Octubre, Madrid, Spain; 4 Hospital Universitario Vall D’Hebron, Barcelona, Spain; 5 Paediatric Intensive Care Unit, Hospital General Universitario Gregorio Marañón, Calle Doctor Castelo 47, 28007 Madrid, Spain; 6 Hospital Regional Universitario de Málaga, Málaga, Spain; 7 Hospital Universitario Sant Joan de Deu, Esplugues de Llobregat, Spain; 8 Hospital Universitario de Burgos, Burgos, Spain; 9 Hospital Universitario Virgen de La Salud, Toledo, Spain; 10 Hospital Universitario Ramón Y Cajal, Madrid, Spain; 11 Clínica Universidad de Navarra, Pamplona, Spain; 12 Complejo Asistencial Universitario de León, León, Spain; 13 Complejo Hospitalario Universitario de Santiago, Santiago de Compostela, Spain; 14 Hospital General Universitario de Albacete, Albacete, Spain; 15 Hospital General Universitario de Alicante, Alicante, Spain; 16 Hospital Universitario Marques de Valdecilla, Santander, Spain; 17 Hospital Universitario de Cruces, Barakaldo, Spain; 18 Hospital Universitario Joan XXIII, Tarragona, Spain; 19 Hospital Universitario Puerta del Mar, Cádiz, Spain; 20 Hospital Universitario Quirónsalud Madrid, Pozuelo de Alarcón, Spain; 21 Hospital Universitario Virgen del Rocío, Sevilla, Spain; 22 Hospital Universitario Virgen de La Macarena, Sevilla, Spain; 23 Parc Tauli Hospital Universitari, Sabadell, Spain

**Keywords:** SARS-CoV-2, Pediatric multisystem inflammatory syndrome temporally associated with COVID-19, Kawasaki disease, Toxic shock syndrome, Children, Critical care, Shock

## Abstract

**Background:**

Multisystem inflammatory syndrome temporally associated with COVID-19 (MIS-C) has been described as a novel and often severe presentation of SARS-CoV-2 infection in children. We aimed to describe the characteristics of children admitted to Pediatric Intensive Care Units (PICUs) presenting with MIS-C in comparison with those admitted with SARS-CoV-2 infection with other features such as COVID-19 pneumonia.

**Methods:**

A multicentric prospective national registry including 47 PICUs was carried out. Data from children admitted with confirmed SARS-CoV-2 infection or fulfilling MIS-C criteria (with or without SARS-CoV-2 PCR confirmation) were collected. Clinical, laboratory and therapeutic features between MIS-C and non-MIS-C patients were compared.

**Results:**

Seventy-four children were recruited. Sixty-one percent met MIS-C definition. MIS-C patients were older than non-MIS-C patients (*p* = 0.002): 9.4 years (IQR 5.5–11.8) vs 3.4 years (IQR 0.4–9.4). A higher proportion of them had no previous medical history of interest (88.2% vs 51.7%, *p* = 0.005). Non-MIS-C patients presented more frequently with respiratory distress (60.7% vs 13.3%, *p* < 0.001). MIS-C patients showed higher prevalence of fever (95.6% vs 64.3%, *p* < 0.001), diarrhea (66.7% vs 11.5%, *p* < 0.001), vomits (71.1% vs 23.1%, *p* = 0.001), fatigue (65.9% vs 36%, *p* = 0.016), shock (84.4% vs 13.8%, *p* < 0.001) and cardiac dysfunction (53.3% vs 10.3%, *p* = 0.001). MIS-C group had a lower lymphocyte count (*p* < 0.001) and LDH (*p* = 0.001) but higher neutrophil count (*p* = 0.045), neutrophil/lymphocyte ratio (*p* < 0.001), C-reactive protein (*p* < 0.001) and procalcitonin (*p* < 0.001). Patients in the MIS-C group were less likely to receive invasive ventilation (13.3% vs 41.4%, *p* = 0.005) but were more often treated with vasoactive drugs (66.7% vs 24.1%, *p* < 0.001), corticosteroids (80% vs 44.8%, *p* = 0.003) and immunoglobulins (51.1% vs 6.9%, *p* < 0.001). Most patients were discharged from PICU by the end of data collection with a median length of stay of 5 days (IQR 2.5–8 days) in the MIS-C group. Three patients died, none of them belonged to the MIS-C group.

**Conclusions:**

MIS-C seems to be the most frequent presentation among critically ill children with SARS-CoV-2 infection. MIS-C patients are older and usually healthy. They show a higher prevalence of gastrointestinal symptoms and shock and are more likely to receive vasoactive drugs and immunomodulators and less likely to need mechanical ventilation than non-MIS-C patients.

## Background

In January 2020 a novel coronavirus (SARS-CoV-2) was described in Wuhan, China. This virus produces the coronavirus disease 2019 (COVID-19), and its rapid spread has led to the declaration of a global health emergency and pandemic by the World Health Organization [1, 2]. The infection affects adults more frequently than children and the clinical manifestations of the infection are generally less severe in pediatric patients than in adults [3–7].

To date, the scarce literature on pediatric patients with SARS-CoV-2 infection shows that it most commonly affects short-age children in the form of respiratory problems [3, 4, 6–10], although severe respiratory symptoms are significantly more frequent in adults than in children [11]. Indeed, only a low proportion of pediatric patients with SARS-CoV-2 infection require intensive care [12, 13], and mortality is lower in children than in adults [12].

By the end of April 2020 and the beginning of May, several scientific societies reported a new clinical presentation related to SARS-CoV-2 infection [14–17]. This syndrome, known as *multisystem inflammatory syndrome temporally associated with COVID-19* (MIS-C), is characterized by fever, abdominal pain, gastrointestinal and cutaneous symptoms, and hemodynamic alterations. As described in these reports, MIS-C has similar features to those of Kawasaki disease (KD), toxic shock syndrome (TSS), bacterial sepsis, and macrophage-activation syndrome. To date, several publications have reported clusters of patients with different severity in the UK [10, 18–21], Italy [22], France [23–25], and the USA [26–28]. In Spain, 5 cases of children presenting with suspected acute abdomen and SARS-CoV-2 infection were initially reported in a single-center study [29]. General data of patients with SARS-CoV-2 admitted to Spanish PICUs have been published with no differentiation of those presenting with MIS-C in a previous report [30].

Although the presence of KD symptoms has been previously described in patients with infections by other coronaviruses [31, 32], the evidence provided on KD in pediatric patients with SARS-CoV-2 in the scientific literature is very limited.

The purpose of this prospective multicenter registry carried out in Spain, is to compare the cases of pediatric patients with SARS-CoV-2 infection and clinical symptoms consistent with MIS-C to those with SARS-CoV-2 infection not fulfilling MIS-C criteria.

## Methods

The Spanish Society of Pediatric Intensive Care promoted the creation of a multicentric prospective registry of pediatric patients with SARS-CoV-2 infection admitted to PICUs in Spain. The Registry was approved by the Institutional Review Board of the coordinating center (Hospital General Universitario Gregorio Marañón). The Registry is fed by 47 PICUs, which account for more than 90% of the PICUs of the Spanish health system. Researchers at each participating PICU were responsible for identifying those who fulfilled inclusion criteria among every admitted patient during the study period. Written informed consent was obtained from patients or their parents before data collection. Data about patients who met inclusion criteria were collected by an electronic data collection form and entered into the Registry by researchers at each site. Data were collected between the 1st of March 2020 and the 15th of June 2020. Partial data from patients included in the study have been previously published as case series elsewhere [29, 30].

### Inclusion criteria

All patients younger than 18 years admitted to a PICU with a diagnosis of SARS-CoV-2 infection were included. Patients who met the case definition for MIS-C according to the criteria of the Royal College of Paediatrics and Child Health (RCPCH) [14] were also included, even lacking a confirmed microbiological diagnosis of SARS-CoV-2 infection.

A case of MIS-C was defined as persistent fever (for longer than 4 days), laboratory data showing inflammation (neutrophilia, lymphopenia, or elevated C-reactive protein) and evidence of organ dysfunction (as described in the variables section) with other additional symptoms such as cutaneous–mucosal or abdominal involvement. Patients with other infections explaining their symptoms were excluded. Polymerase chain reaction (PCR) testing for SARS-CoV-2 in patients with MIS-C may be positive or negative [14].

As cases consistent with MIS-C may have been overlooked before the definition of this entity was published, researchers at every participating PICU were asked to perform a retrospective review of all admissions since the Registry's inception. None of the participating PICUs identified any patient who met the established criteria and had not been previously included in the Registry.

### Variables collected

Collected data included the date of admission to and discharge from PICU, demographic variables; any medical history of interest; and the main reason for PICU admission. Other data included were clinical diagnosis within the first 24 h of admission, severity scores (PRISM III) and multi-organ failure scores (P-SOFA), and other clinical and analytical parameters recorded on admission and during the hospital stay. A clinical diagnosis of shock was established in the presence of arterial hypotension with mean blood pressure values below the 5th percentile of the reference values for age, need for vasoactive therapy to maintain normal blood pressure, or presence of signs of hypoperfusion despite adequate fluid resuscitation. Acute cardiac dysfunction was defined as the appearance of any of the following echocardiographic alterations: global or segmental contractility alterations, ventricular dilatation, reduced ejection fraction and/or presence of pericardial effusion.

Additional data included the presence or absence of the following symptoms: fever, cough, respiratory distress, odynophagia, rhinorrhea, diarrhea, nausea, vomiting, refusal to eat, headache, irritability, altered level of consciousness, seizures, fatigue, myalgia or abdominal pain.

Additionally, symptoms consistent with hyperinflammatory syndromes such as KD and TSS were recorded in patients who met the MIS-C case definition. Possible symptoms of hyperinflammatory syndromes included fever; persistent fever (more than 4 days of duration); ocular manifestations such as non-exudative conjunctivitis; oral mucosa lesions; cutaneous manifestations (palmar or plantar erythema, edema, swelling, desquamation, erythroderma, or polymorphic erythema in the hands and feet); cervical lymphadenopathy; arterial hypotension; renal, hematological, or hepatic involvement; presence of Acute Respiratory Distress Syndrome (ARDS); neurological involvement, or soft tissue necrosis. The presence of ARDS was defined following the Pediatric Acute Lung Injury Consensus Conference criteria [33]. For renal involvement, the definition employed was an increase in creatinine levels of double the normal limits for the age of the patient or twice as baseline creatinine levels. The considered definition of hematologic involvement included the presence of coagulopathy (aPTT or PT above normal limits) or thrombocytopenia (< 100,000 platelets/mcl). Liver involvement was defined as an increase in transaminase or bilirubin levels twice above baseline or normal values for the age of the patient.

Laboratory data on admission and during hospital stay were also collected, including hemoglobin, leukocyte and platelet counts, coagulation tests, D-dimer, creatinine, urea, liver function markers, LDH, and ions. Inflammatory markers such as C reactive protein (CRP), procalcitonin (PCT), and IL-6 were recorded. In patients with hyperinflammatory symptoms, NT-ProBNP, and serum troponin levels were also included. Microbiologically confirmed diagnosis of SARS-CoV-2 infection (PCR and serology) was also recorded. PCR testing was performed on nasopharyngeal swab samples. The microbiological tests were carried out according to the established practice at each participating center.

Besides, antibiotic, antiviral, and immunomodulatory therapies administered during PICU stay, respiratory and cardiovascular support (administration of vasoactive drugs and/or ECMO) or need for renal replacement therapy were obtained.

### Statistical analysis

Data were entered into a database that was analyzed using the SPPS statistical package (IBM SPSS Statistics, Version 25.0. Armonk, NY: IBM Corp). Continuous quantitative variables were expressed as median values and interquartile ranges as measures of central tendency. Comparison of continuous quantitative variables was performed using the Wilcoxon rank-sum test. Comparison of proportions between subgroups of patients was carried out by Chi-squared test and Fisher's test when the first method could not be used due to the small number of cases. Heatmap and dendrogram were performed using Jaccard distance and Jaccard index to describe clusters of co-occurring symptoms. Dendrogram was built using Jaccard distance as metric and complete linkage as adjustment method. Heatmap and dendrogram were drawn using Datagraph for MacOS version 4.5.1 (Visual Data Tools Inc. Chapel Hill, NC). When some data were missing, the number of patients whose data were available for each variable is stated.

## Results

Forty-seven PICUs, including 1st, 2nd and 3rd level of care, from every region of Spain participated in the Registry. Between March 1st and June 15th, 2020, a total of 74 children admitted to the participating PICUs were included in the Registry, with ages ranging from 15 days to 16.5 years. Forty-five patients (61%) were male, and 52 (70.3%) had no previous disease. Forty-five patients (61%) met the case definition for MIS-C proposed by the RCPCH.

SARS-CoV-2 infection was confirmed microbiologically in 61 of 74 patients (82.4%). Viral RNA was detected by PCR in 44 of 74 patients (59.5%). Serologic tests were done in 37 patients (50%) being positive in 27 of them (73%). The diagnosis was not confirmed microbiologically in thirteen patients (17.6%) with high suspicion of SARS-CoV-2 infection (according to clinical, epidemiological, or radiologic findings) or meeting MIS-C criteria. Among patients not presenting with MIS-C, viral RNA was detected using PCR in 26 of 29 (89.7%) while in the MIS-C group, PCR was positive in 18 out of 45 (40%) (*p* < 0.001). Seventeen of the 27 (63%) patients with negative PCR testing in the MIS-C group had serological confirmation of SARS-CoV-2 infection.

**Fig. 1:**
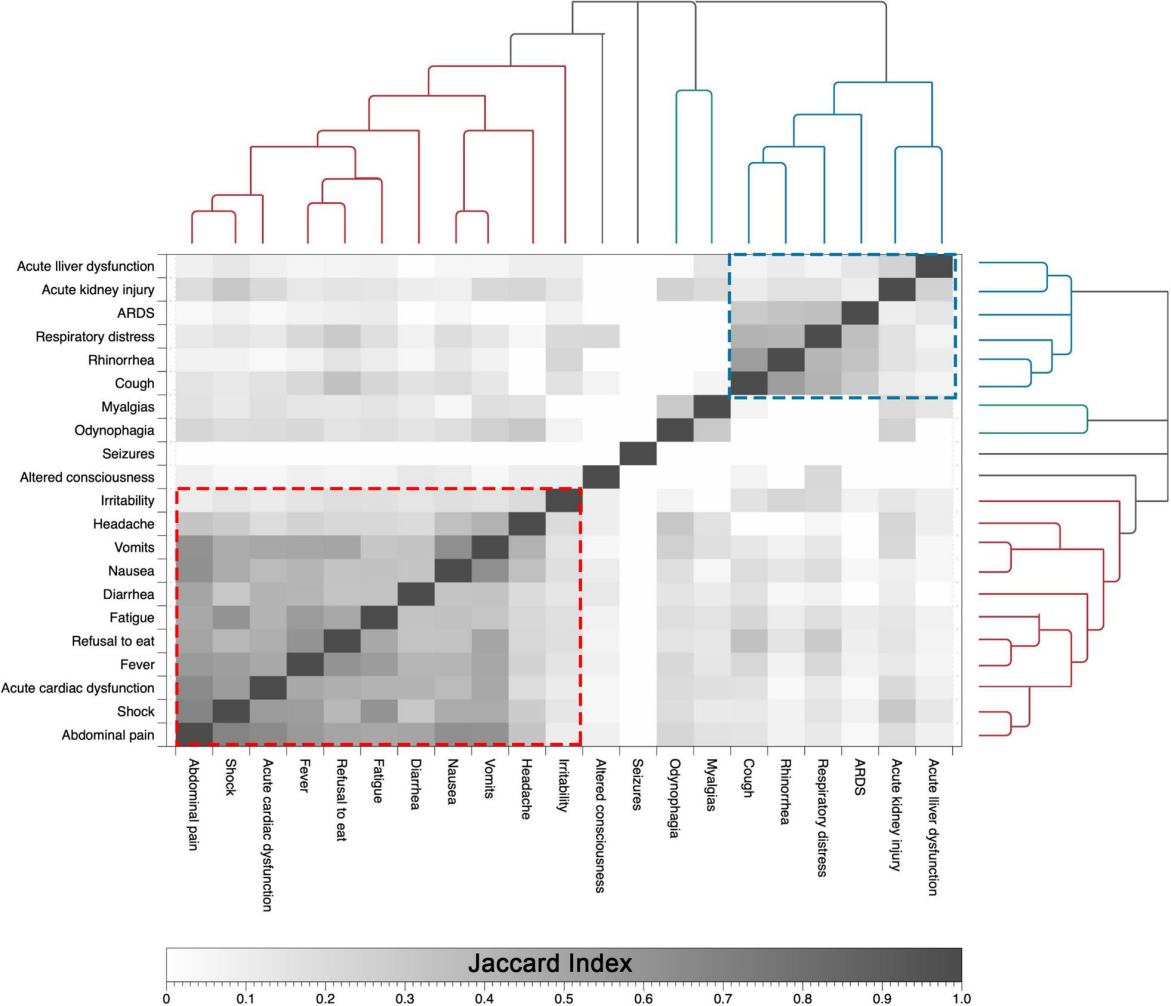
Heatmap and dendrogram describing clusters of co-occurring symptoms. Jaccard index was used to describe co-occurrence. Jaccard index is the ratio of the number of times two symptoms occur together divided by the number of times either of them appears. Jaccard index ranges from 0 (symptoms never appear together) to 1 (symptoms always appear together). Red square includes those symptoms clustered in patients presenting with MIS-C features. Blue square includes those symptoms co-occurring in patients presenting with respiratory disease. *ARDS* Acute respiratory distress syndrome

**Fig. 2:**
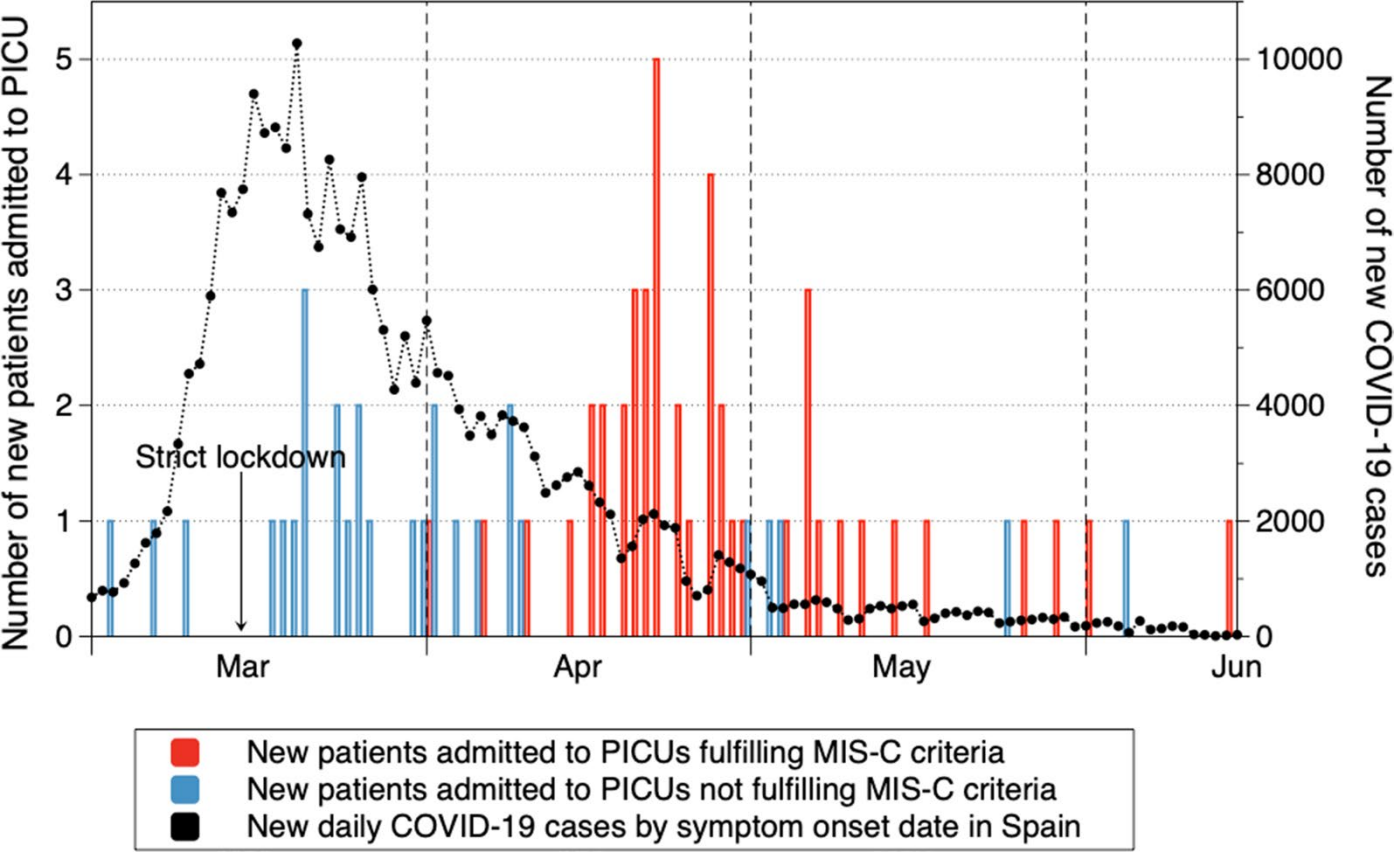
Frequency of new admissions in PICUs participating in the Registry by date of admission. Patients fulfilling MIS-C criteria and not fulfilling MIS-C criteria are marked with different colors. Black dots represent number of new daily cases of COVID-19 in Spain according to symptom onset. Strict lockdown was imposed in Spain on 15 March, 2020

Table 1 compares the baseline characteristics, symptoms prior to admission, and clinical diagnoses established within the first 24 h of admission of the group of patients meeting the case definition for MIS-C vs the remainder of patients with SARS-CoV-2 infection admitted to the PICU. The group of patients with MIS-C had an older age and higher weight. The proportion of patients with a previous medical history of interest was lower in the group of patients meeting MIS-C criteria. Respiratory symptoms were less frequent in the MIS-C patients, whereas the incidence of gastrointestinal symptoms and fatigue was higher in this group. The MIS-C group presented a higher prevalence of symptoms of shock and acute cardiac dysfunction. Clusters of symptoms were identified using heatmap and dendrogram analysis (Fig. 1). Figure 2 shows the count of new SARS-CoV-2 positives admitted to the PICU every day separating patients with MIS-C from the remainder of patients. Table 2 displays the incidence of KD and TSS symptoms in the 45 patients with MIS-C.

**Table 1:** Clinical manifestations of SARS-COV-2 in critical pediatric patients

	Total patients (*N* = 74)	Patients with MIS-C (*N* = 45)	Patients without MIS-C (*N* = 29)	*p*
Gender (male) *n*/*N* (%)	46/74 (62.2)	39/45 (66.7)	16/29 (55.2)	0.320
*Age (years) Median (IQR)*	8.1 (3–11.5)	9.4 (5.5–11.8)	3.4 (0.4–9.4)	**0.002**
0–5 years *n*/*N* (%)	30 (40.5)	12 (26.7)	18 (62.1)	**0.003**
6–12 years *n*/*N* (%)	33 (44.6)	27 (60)	6 (20.7)	
> 13 years *n*/*N* (%)	11 (14.9)	6 (13.3)	5 (17.2)	
Weight (kg) Median (IQR)	29.5 (15–43.5)	36 (22.5–50)	15.5 (6.5–32.5)	**< 0.001**
Previously healthy *n*/*N* (%)	52/74 (70.3)	37/45 (82.2)	15/29 (51.7)	**0.005**
PRISM III Median (IQR)	7 (4–13.5)	7 (5–14)	7 (3–13)	0.544
p-SOFA Median (IQR)	4 (2–6)	4 (3–6)	3 (1–5)	0.135
*Symptoms prior to PICU admission*				
Fever *n*/*N* (%)	61/73 (83.6)	43/45 (95.6)	18/28 (64.3)	**< 0.001**
Cough *n*/*N* (%)	26/73 (35.6)	12/45 (26.7)	14/28 (50)	**0.043**
Respiratory distress *n*/*N* (%)	23/73 (31.5)	6/45 (13.3)	17/28 (60.7)	**< 0.001**
Odynophagia *n*/*N* (%)	12/68 (17.6)	9/43 (20.9)	3/25 (12)	0.352
Rhinorrhea *n*/*N* (%)	11/71 (15.5)	2/43 (4.7)	9/28 (32.1)	**0.002**
Diarrhea *n*/*N* (%)	33/71 (46.5)	30/45 (66.7)	3/26 (11.5)	**< 0.001**
Nausea *n*/*N* (%)	22/53 (41.5)	18/30 (60)	4/23 (17.4)	**0.002**
Vomits *n*/*N* (%)	38/71 (53.5)	32/45 (71.1)	6/26 (23.1)	**< 0.001**
Refusal to eat *n*/*N* (%)	48/69 (69.6)	33/43 (76.7)	15/26 (57.7)	0.096
Headache *n*/*N* (%)	16/68 (23.5)	13/44 (29.5)	3/24 (12.5)	0.113
Irritability *n*/*N* (%)	15/71 (21.8)	9/45 (20)	6/26 (23.1)	0.760
Altered consciousness *n*/*N* (%)	5/72 (6.9)	2/45 (4.4)	3/27 (11.1)	0.281
Seizures *n*/*N* (%)	1/72 (1.4)	1/45 (2.2)	0/27 (0)	0.435
Fatigue *n*/*N* (%)	38/69 (55.1)	29/44 (65.9)	9/25 (36)	**0.016**
Myalgias *n*/*N* (%)	8/67 (11.9)	7/43 (16.3)	1/24 (4.2)	0.143
*Diagnoses within the first 24 h of admission*				
ARDS *n*/*N* (%)	11/74 (14.9)	3/45 (6.7)	8/29 (27.6)	**0.014**
Shock *n*/*N* (%)	42/74 (56.8)	38/45 (84.4)	4/29 (13.8)	**< 0.001**
Acute kidney injury *n*/*N* (%)	12/74 (16.2)	9/45 (20)	3/29 (10.3)	0.271
Acute cardiac dysfunction *n*/*N* (%)	27/74 (36.5)	24/45 (53.3)	3/29 (10.3)	**0.001**
Acute liver dysfunction *n*/*N* (%)	14/74 (18.9)	11/45 (24.4)	3/29 (10.3)	0.131

Comparison of patients with MIS-C and without MIS-C. Quantitative values are expressed as median values and interquartile range. Qualitative variables are expressed as number of cases with respect to the total number of cases in each group and percentages

*IQR* Interquartile rank, *p-SOFA* pediatric sequential organ failure, *PRISM III* Pediatric risk of mortality score, *ARDS* Acute respiratory distress syndrome. The criteria used to define diagnostic variables within the first 24 h of admission are defined in the Materials and Methods section

**Table 2:** Frequency of characteristic symptoms of other hyperinflammatory syndromes as KD and TSS in patients with MIS-C

Symptom	Number of patients	%
Fever	43/45	95.6
Arterial hypotension for the age	41/45	91.1
Abdominal pain	40/45	90.9
Cutaneous manifestations	31/45	68.9
Prolonged fever (> 4 days)	27/45	60
Hematologic alterations	26/45	68.4
Ocular manifestations	18/45	40
Liver involvement	14/45	31.8
Alterations in the oral mucosa	10/45	27
Renal involvement	8/45	17.8
Neurologic involvement	6/45	13.6
Soft tissue necrosis	2/45	5.4
Lymphadenopathy	2/45	5.4
ARDS	2/45	4.7

*ARDS* Acute respiratory distress syndrome. The criteria used to define the different variables are defined in the Materials and Methods section

**Fig. 3:**
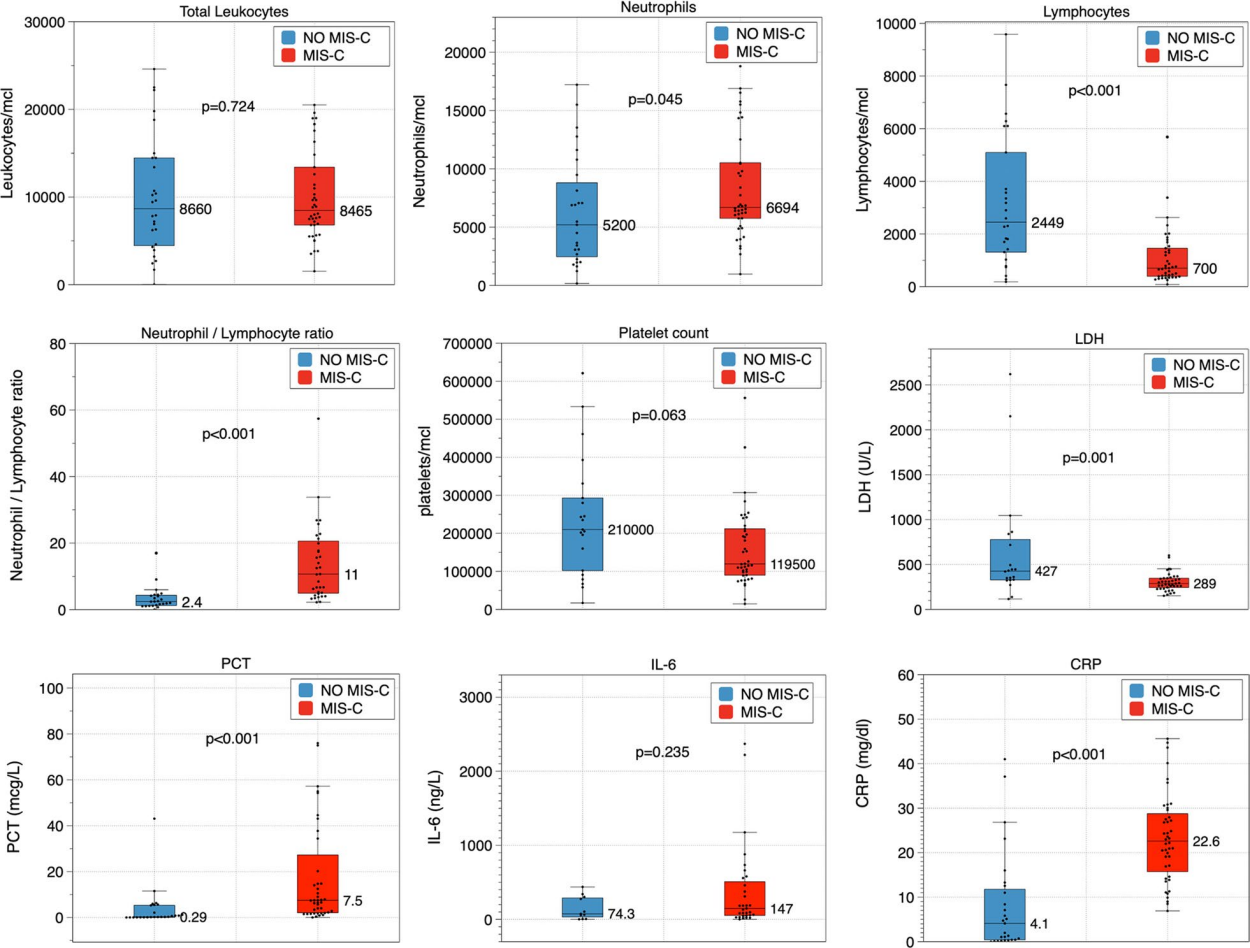
Laboratory findings of patients fulfilling and not fulfilling MIS-C criteria

Figure 3 compares the laboratory parameters of patients with and without MIS-C. Patients with MIS-C exhibited lower levels of lymphocytes and LDH, higher levels of CRP and PCT, neutrophils, and a higher lymphocyte/neutrophil ratio. Troponin-T was determined in 34 of the 45 MIS-C patients (75.6%) with a median value of 55.4 ng/L (IQR 13.3–204.7 ng/L) (normal value < 19 ng/L) [34] and NT-ProBNP was determined in 22 patients with MIS-C (48.9%) with a median value of 5,532 pg/ml (IQR 1582–12,783 pg/ml) (normal value < 450 pg/ml) [35]. Twenty-two patients in the MIS-C group (48.9%) exhibited echocardiographic signs of ventricular dysfunction. Coronary arteries abnormalities were reported in 3 patients in the MIS-C group (6.7%): two patients had a homogeneously dilated anterior descendent artery along its entire length, and one patient had a dilated left coronary artery with an aneurism in the anterior descendent artery.

Support measures and antibiotic, antiviral, and immunomodulatory therapies administered during PICU hospitalization are shown in Table 3. Invasive and noninvasive mechanical ventilation were less used in patients with MIS-C, whereas the use of antibiotics and vasoactive and immunomodulatory therapies was more common in these patients.

**Table 3:** Support measures and pharmacological treatments administered to patients admitted for SARS-COV-2

	Total patients (*N* = 74)	Patients with MIS-C (*N* = 45)	Patients without MIS-C (*N* = 29)	*p*
*Support measures. n/N (%)*				
Oxygen therapy	56/74 (79.7)	36/45 (80)	20/29 (69)	0.215
HFO	26/74 (35.1)	14/45 (31.1)	12/29 (41.4)	0.379
NIV	9/74 (12.2)	2/45 (4.4)	7/29 (24.1)	**0.026**
MV	18/74 (24.3)	6/45 (13.3)	12/29 (41.4)	**0.005**
Neuromuscular blockade	12/74 (16.2)	3/45 (6.7)	9/29 (31)	**0.009**
Ventilation in prone position	9/74 (12.2)	1/45 (2.2)	8/29 (27.6)	**0.002**
V-V-ECMO	1/74 (1.4)	0/45 (0)	1/29 (3.4)	0.394
A-V-ECMO	1/74 (1.4)	1/45 (2.2)	0/29 (0)	1
NOi	3/74 (4.1)	0/45 (0)	3/29 (10.3)	0.057
CRRT	0/74 (0)	0/45 (0)	0/29 (0)	-
Transfusion of hemoderivatives	14/74 (18.9)	5/45 (11.1)	9/29 (31)	**0.034**
Vasoactive drugs	37/74 (50)	30/45 (66.7)	7/29 (24.1)	**< 0.001**
*Pharmacological therapies n/N (%)*				
Antibiotic	65/74 (87,8)	42/45 (93.3)	23/29 (79.3)	**0.020**
Lopinavir–ritonavir	30/74 (40.5)	18/45 (40)	12/29 (41.4)	0.857
Remdesivir	5/74 (6.8)	0/45 (0)	5/29 (17.2)	**0.006**
Hydroxychloroquine	43/74 (58.1)	25/45 (55.6)	18/29 (62.1)	0.550
Corticosteroids	49/74 (66.2)	36/45 (80)	13/29 (44.8)	**0.003**
Immunoglobulins	25/74 (33.8)	23/45 (51.1)	2/29 (6.9)	**< 0.001**
Tocilizumab	17/74 (23)	11/45 (24.4)	6/29 (20.7)	0.871

Comparison of patients with MIS-C and without MIS-C

*HFO* high-flow rate oxygen, *NIV* noninvasive ventilation, *MV* invasive mechanical ventilation, *V-V-ECMO* veno-venous extracorporeal membrane oxygenation, *V-A ECMO* veno-arterial extracorporeal membrane oxygenation, *NOi* Inhaled nitric oxygen, *CRRT* continuous renal replacement therapy

Among 18 patients requiring intubation in 11 (61.1%) the main reason for intubation was respiratory failure while in 7 (38.9%) it was due to hemodynamic failure. Patients requiring invasive ventilation were younger (2.9 years [IQR 0.4–9.5]) than those who did not (9.1 years [IQR 4.2–11.9]), *p* = 0.007. They also had higher PRISM-III score (13 points [IQR 8–16]) than non-ventilated patients (6.5 points [IQR 4–9.3]), *p* = 0.005, and higher p-SOFA scores (6 points [IQR 4–10.5] vs 3 points [IQR 2–5]), *p* < 0.001. On the contrary, patients requiring ventilation had lower CRP levels (7.1 mg/dl [IQR 0.9–21.6]) than non-ventilated patients (20.5 mg/dl [IQR 10.3–27.6]), *p* = 0.017. Among 18 patients requiring invasive ventilation, 15 (83.3%) had radiologic alterations in the chest x-ray.

Radiologic alterations in the chest-x-ray were present in 20 patients (55.6%) with MIS-C features and in 27 patients (93.1%) without (*p* = 0.001). The existence of an abnormal chest-x-ray was observed in 36 of 44 patients (81.8%) with positive PCR for SARS-CoV-2 in the nasopharyngeal swab samples and in 16 of 30 patients (53.3%) of patients with negative nasopharyngeal swab SARS-CoV-2 PCR (*p* = 0.008). Twenty-two patients of 74 (29.7%) showed normal chest-x-ray at admission. Patients with normal chest-x-ray had higher PCT 7.9 mcg/L (3.1–55.7) than patients with chest-x-ray alterations (1.9 mcg/L [0.3–7.4]), *p* = 0.006.

Thirty-seven patients required vasoactive drugs, among them, 15 (40%) showed conserved cardiac function. Noradrenaline and dopamine were used in more patients in the MIS-C group (35.5% and 25.8%) compared to the non-MIS-C group (7.7% and 4%), *p* = 0.024 and *p* = 0.033, respectively. Fluid balance per kilogram during the first day of admission was 9.2 ml/kg (IQR − 7.1 to 28) in the group of patients without MIS-C and 21.8 ml/kg (IQR 0.2–29.7) *p* = 0.450.

The administration of antiviral treatments was similar in the two groups, except for remdesivir, which was not used in patients with MIS-C.

As of June 15, 2020, 65 of the 74 patients (87.8%) included in the Registry had been discharged from the PICU. The length of stay in patients with MIS-C was 5 days (2.5–8 days) versus 6.5 days (3.3–10.8 days) in the other group (*p* = 0.523). The patient who required VA-ECMO was weaned and discharged to the ward. Three patients included in the registry died (4%), all of them belonged to the group of patients not presenting with MIS-C. All deceased patients previously had serious illnesses: one of them had a Niemann–Pick disease, and the other two had received hematopoietic stem cell transplantation due to acute lymphoblastic leukemia and to severe immunodeficiency. Deceased patients developed severe respiratory distress, one of them died while he was on VV-ECMO support due to massive pulmonary thromboembolism.

## Discussion

In general terms, the clinical manifestations of SARS-CoV-2 infection are less severe in pediatric patients than in adults. The study published by Dong et al. revealed that only 6% of the more than 2,000 pediatric patients included, developed severe clinical symptoms [7], and only a small proportion needed intensive care. Respiratory problems are less frequent in children than in adults [3].

The occurrence of severe systemic hyperinflammatory symptoms probably associated with SARS-CoV-2 infection in children has raised concerns among scientific societies [14–17]. This syndrome, which symptoms mimic those of sepsis, KD or TSS, has been described in different studies [18–29]. The information available on the most severe manifestation of SARS-CoV-2 in children is also very limited [12, 36]. Our multicentric Registry identified the differences between PICU patients with symptoms of hyperinflammatory syndrome and those with SARS-CoV-2 infection without symptoms of hyperinflammation. To date large series of pediatric patients suffering severe manifestations of SARS-CoV-2 infection are lacking, and our study includes an important number of them. It is the first describing characteristics of SARS-CoV-2 infection comparing the patients presenting with multisystem inflammatory symptoms with those without.

Whereas the most of COVID-19 patients in the studies performed by Shekerdemian et al. and Sachdeva et al. in the USA and Canada [12, 36] had respiratory symptoms, inflammatory syndromes were more frequent in our series. This difference may be explained by the fact that Spain is in a more advanced stage of the pandemic, as no cases of this type were recorded in our Registry during the initial stages. Several studies in different regions have shown a delay between the peak of incidence of COVID-19 cases and the rise in the number of MIS-C cases [10, 21, 22, 27, 28]. In the same vein, the presence of previous comorbidities was higher in the sample of Shekerdemian et al. (above 80%), whereas previous comorbidities in our sample were only identified in patients without systemic inflammatory symptoms, who were prevailingly hospitalized during the first weeks of operation of the Registry. Many published studies point out that most patients presenting with MIS-C are previously healthy [19–21, 24, 26–28].

In total, 75% of pediatric patients with MIS-C were older than 6 years. This finding contrasts with KD, which is more frequent in children less than 5 years old [37]. These findings are consistent with those observed in the studies published by Whittaker et al. and Verdoni et al. where MIS-C patients are compared with previous cohorts of KD patients showing older age [19, 22].

Cluster analysis has identified two big groups of patients in our cohort, those presenting with fever, shock, acute cardiac dysfunction, and gastrointestinal symptoms (the group of patients with MIS-C features), and a second group presenting with respiratory symptoms including ARDS that often associates also acute liver and kidney dysfunction (the group of patients with a more classical presentation of COVID-19).

In patients with MIS-C, gastrointestinal symptoms are more frequent than respiratory symptoms, whereas patients with SARS-CoV-2 typically show respiratory symptoms. This finding contradicts the data reported by Shekerdemian et al., who reported a very low incidence of gastrointestinal problems (2%) [12]. In our series, gastrointestinal symptoms were uncommon in patients without MIS-C (4% of these patients have diarrhea), whereas more than 60% of patients with inflammatory syndromes exhibited gastrointestinal symptoms. This is in line with the previously described MIS-C case series, in which abdominal symptoms as abdominal pain, diarrhea, nausea and vomiting are present in most patients [10, 18–24, 26–28].

Fever is the most frequent symptom in patients with MIS-C, with 100% of patients developing a fever, whereas a third of the patients without MIS-C do not present a fever. Almost the totality of patients with MIS-C of our series exhibited arterial hypotension. It is worth noting that we only analyzed PICU patients. Therefore, there may have been patients with milder MIS-C symptoms who did not develop hemodynamic alterations and did not require intensive care as described in studies that include patients not requiring intensive care [19, 20, 22, 23, 27, 28].

As mentioned above, the clinical-laboratory manifestations of MIS-C mimic those of KD and TSS, and a high proportion of patients may meet the diagnostic criteria for both diseases [37, 38]. However, some of the typical symptoms of these diseases were very uncommon in our series, especially the typical cervical adenopathy of KD or the oral mucosa and lips lesions (only present in 1 out of 4 patients of our series) [37]. Some studies have described how the presence of cutaneous manifestations in children with MIS-C might vary according the age, being less frequent in older patients [27, 28].

Concerning the laboratory parameters, the MIS-C patients presented severe inflammation, with very elevated levels of acute-phase reactants (CRP and PCT), exceeding those of SARS-CoV-2 patients without MIS-C. Although absolute leukocyte counts were not very elevated and were similar in the two groups, patients with MIS-C exhibited severe lymphopenia, with a high neutrophils/lymphocyte ratio and a low platelet count. Special attention needs to be paid to the fact that LDH was lower in patients with MIS-C as compared to those without MIS-C, since elevated LDH levels may be associated with hemolysis or myocardial damage. The clinical interpretation of this finding is challenging. The analytical findings in our series were similar to those previously reported, i.e., lymphopenia without significant leukocytosis, thrombocytopenia, and high CRP and PCT [18–22, 27, 28].

Hyperinflammatory state has been previously reported in adults presenting with COVID-19. This hyperinflammatory state has been related to disease severity and the need for mechanical ventilation and has been described in the context of classic COVID-19 syndrome presenting with bilateral pneumonia [39, 40]. In our study, contrary to the studies in adults, patients presenting with hyperinflammatory features such as elevated CRP, showed lower prevalence of chest x-ray abnormalities and lesser need of mechanical ventilation. Our study points out differences regarding hyperinflammatory states related to SARS-CoV-2 infection in children as compared to those described in adults. In adults, hyperinflammation is more frequent in the context of COVID-19 bilateral pneumonia and in children in patients with mild or absent respiratory symptoms presenting gastrointestinal symptoms and shock fulfilling MIS-C criteria.

Several authors have proposed a mechanism of immune dysregulation underlying MIS-C cases. In children, increased antibodies against receptor binding domain of SARS-CoV-2 have been described in patients with MIS-C compared with patients with COVID-19 without hyperinflammatory features [41]. Activation of neutrophils and monocytes has been also described in the acute phase of pediatric patients with MIS-C with normalization in the resolution and convalescent phases of the disease [42]. In adults with severe COVID-19, hyperinflammation and abnormal activation of different cell lineages have also been described widely [43]. However, it remains unclear why in children, SARS-CoV-2 related hyperinflammatory states seems to affect more frequently cardiovascular and digestive organs instead of the lungs as seen in adults.

With regard to the treatments administered, most MIS-C patients admitted to the PICU required vasoactive therapy, whereas this therapy was less frequent in patients without this MIS-C. There was a high proportion of MIS-C patients requiring vasoactive drugs with conserved cardiac function and use of noradrenaline was also higher in this group. These facts might point out an important element of vasoplegia in MIS-C patients.

In contrast with the use of vasoactive drugs, the need of mechanical ventilation was higher amongst those patients without MIS-C. In overall terms, the use of mechanical ventilation nearly reached 30%, which is similar to the percentage reported in studies about PICU admissions including children with SARS-CoV-2 related diseases [12, 36]. In our population of patients with MIS-C, the use of mechanical ventilation was infrequent (below 15%) as described by Dufort et al. in New York State [28], and lower to the rates described in studies from other regions as U.S.A., U.K. and France where more than 30% of patients with MIS-C needed mechanical ventilation.

As to pharmacological treatments, most patients with MIS-C included in the Registry received antibiotic therapy. The use of immunomodulatory and corticosteroid treatments was also higher in the group of patients with MIS-C. No differences were observed in the use of antiviral treatments, although remdesivir was not administered to any of the patients with MIS-C. Remdesivir availability was limited in Spain during this phase of the pandemic. The distribution of remdesivir was subject to request and authorization by the ministry of health and administration in children was considered only in the context of a clinical trial. Remdesivir administration was requested in several patients in the MIS-C group but delay related to approval and favorable course in most MIS-C patients probably determined the difference in its use between groups. In view of the scant evidence available on the effectiveness of antiviral therapies in the treatment of SARS-CoV-2, further studies should be conducted to assess the efficacy of the immunomodulators used to treat similar symptoms to those described above (such as steroids and immunoglobulins) to attenuate the inflammatory mechanisms involved in the disease [44]. Treatments used in patients with MIS-C are similar to those described in studies from other regions [21, 27, 28].

The relationship observed between time and the occurrence of MIS-C cases is worthy of note. All MIS-C patients were admitted to the PICU at least 15 days after the lockdown was imposed in Spain on March 15, 2020. In the stage where the first patients with MIS-C were recorded, in early April, the number of new cases was already decreasing. The simultaneous occurrence of similar symptoms to those described in our series in other European countries [18–24] leads us to foresee an increase in the incidence of this syndrome in more advanced stages of the epidemic in countries where the coronavirus spike occurred some weeks later than in Europe. This added to the fact that PCR was negative in more than half of MIS-C patients but serology was positive for SARS-CoV-2 in a high proportion of them suggests that MIS-C may be caused by an underlying deregulation of the immune system, with the viral infection triggering a hyperinflammatory response rather than being a direct expression of SARS-CoV-2 infection [22].

As to prognosis, the course of MIS-C patients included in our registry was generally favorable, without any mortality. Most patients were discharged to the ward in a few days. Other studies describe similar findings with low mortality in patients with MIS-C (below 3% in all series) [19, 21, 23, 24, 27, 28].

### Limitations

This study has some limitations. First, it is a multicentric study that includes most—but not all—PICUs in Spain. Therefore, some patients with similar symptoms could have not been included in the study. We consider that PICUs not participating in the registry do not represent a specific region or subset of PICUS that might prevent generalizing our results to all Spanish PICUs. Secondly, the new syndrome observed led us to retrospectively review the potential cases that may have been overlooked. Although the participating PICUs reviewed their records, some patients may have not been included. Although it is a prospective Registry, some clinical or laboratory variables were missing. Beside this, each hospital had their own microbiological tests and thus differences on diagnostic precision regarding SARS-CoV-2 infection might be present. As only patients admitted to PICU were included in the registry, we consider that probably our study does not include the whole spectrum of MIS-C and some patients might develop milder manifestations. Finally, the long-term course of patients could not be analyzed, given the short period of time elapsed from the onset of the clinical symptoms and analysis. In this sense, given that KD is one of the most frequent causes of heart disease acquired in childhood, it would be very interesting to describe the incidence of coronary lesions in patients with MIS-C.

## Conclusions

Although SARS-CoV-2 infection is less severe in children than in adults, some pediatric patients may present severe symptoms requiring intensive care. In Spain, most pediatric patients requiring intensive care presented with hyperinflammatory syndrome instead of classic COVID-19 disease with severe respiratory involvement. This syndrome is characterized by fever, hypotension, gastrointestinal symptoms and cutaneous manifestations with elevation of inflammatory markers together with significant lymphopenia, requiring vasoactive therapy. The course of this syndrome is generally favorable. The number of cases of MIS-C was higher after the spike in coronavirus cases in Spain. This, and the fact that it is frequent that MIS-C patients have negative SARS-CoV-2 PCR tests supports a causal link between SARS-CoV-2 and MIS-C in which children immune response dysregulation might be involved. Larger, international, multicentric studies are needed to characterize this syndrome more accurately and establish the optimal treatment. Health policies regarding pediatric intensive care units preparedness for SARS-CoV-2 pandemic should take into account that a high proportion of patients will present with MIS-C. Therefore, specific management protocols should be elaborated, and resource acquisition and distribution should consider the previously described patients characteristics and therapeutic needs.

## Data Availability

The datasets used during the current study are available from the corresponding author on reasonable request.

## Abbreviations

MIS-C: Multisystem inflammatory syndrome temporally associated with COVID-19
PICUs: Pediatric intensive care units
KD: Kawasaki disease
TSS: Toxic shock syndrome
RCPCH: Royal College of Paediatrics and Child Health
IQR: Interquartile range
PCR: Polymerase chain reaction
ARDS: Acute respiratory distress syndrome
CRP: C reactive protein
PCT: Procalcitonin
HFO: High-flow rate oxygen
NIV: Noninvasive ventilation
MV: Mechanical ventilation.
VV-ECMO: Veno-venous extracorporeal membrane oxygenation
VA ECMO: Veno-arterial extracorporeal membrane oxygenation
NOi: Inhaled nitric oxygen
CRRT: Continuous renal replacement therapy
